# Lower Lid Reconstruction Utilizing Auricular Conchal Chondral-Perichondral Tissue in Patients with Neoplastic Lesions

**DOI:** 10.1155/2013/837536

**Published:** 2013-06-20

**Authors:** Pier Camillo Parodi, Fabrizio Calligaris, Fabrizio De Biasio, Giovanna De Maglio, Flavia Miani, Marco Zeppieri

**Affiliations:** ^1^Department of Plastic and Reconstructive Surgery, Azienda Ospedaliero-Universitaria “Santa Maria della Misericordia,” Piazzale S. Maria della Misericordia 15, 33100 Udine, Italy; ^2^Department of Histopathology, Azienda Ospedaliero-Universitaria “Santa Maria della Misericordia,” Piazzale S. Maria della Misericordia 15, 33100 Udine, Italy; ^3^Department of Ophthalmology, Azienda Ospedaliero-Universitaria “Santa Maria della Misericordia,” Piazzale S. Maria della Misericordia 15, 33100 Udine, Italy

## Abstract

*Purpose*. To assess surgical outcomes of lower lid reconstruction surgery using auricular conchal tissue. *Methods*. This prospective study included 20 patients that underwent reconstructive lower lid surgery using autologous auricle chondral-perichondral graft tissue. Auricle tissue was used to provide adequate support and protection with similar conjunctiva tarsal structures on overlying soft tissues in patients with pathologic inferior lid tissue loss requiring reconstructive surgery. Biopsies with histopathology and cytology analysis were taken after 1 year. Cytology analysis using CK19 was used to confirm newly formed conjunctiva overlying the graft. *Results*. All patients showed no graft rejection. Surgical outcomes were generally good, with minimal or no ocular complications. 16 of 20 patients had excellent results, showing good lid symmetry and esthetics, minimal auricular discomfort, patient satisfaction and proper lid function. Surgical outcomes were highly dependent on proper post-op conjunctiva formation. All patients were positive for CK19, thus indicating proper conjunctiva tissue formation. *Conclusions*. Lower lid reconstruction surgery using auricular chondral-perichondral conchal tissue is a good alternative in patients with neoplastic lesions. Autologous chondral-perichondral tissue provides good functional and mechanical support in the reconstructed lid, thus reducing the risks of ectropion and corneal exposure and ensuring a protected ocular surface.

## 1. Introduction

Reconstruction of the lower eyelid is always a challenge due to its anatomical complexity. The eyelid is conventionally divided into an anterior lamella, composed of skin and muscle, and a posterior lamella consisting of the tarsus and palpebral conjunctiva. Reconstructive surgery for full-thickness eyelid defects normally requires graft tissue that is similar to the anatomic structure, which should include the following elements: an outer layer skin surface, an inner layer mucosa, and a suitable semirigid support for stabilizing the rim of the eyelid. One of the two elements is typically reconstructed by means of a host flap, which is capable of providing adequate nourishment for the second grafted element [1].

Matsuo et al. proposed a new surgical method in 1987, which involved using chondroperichondral graft tissue extracted from the donor auricle concha to reconstruct the posterior lamella [2, 3]. The adjacent skin flap was used to restore the anterior lamella. This surgical method did not raise much interest in the literature at that time; however, it represents a valid alternative to traditional methods currently used. The method offers a functional three-dimensional lid reconstruction with tissues similar to those lost to trauma or surgery. Modern day palpebral reconstruction must aim at providing functional anatomical similarity to the healthy lid prior to damage, thus respecting the anatomic complexity. The aim of our study was to assess the safety, efficacy, and long-term surgical outcomes of lower lid reconstruction utilizing auricular conchal tissues in patients with excised neoplastic lid lesions. 

## 2. Methods

### 2.1. Patients

The study included 20 patients (12 males and 8 females; mean age 60.5 ± 5.95; range 48 to 73 years) that underwent eyelid surgery at our Department of Plastic and Reconstructive Surgery from 2002 to 2007. The study was in accordance with the institutional review board, approved by the Ethics Committee, and conforming to Declaration of Helsinki. Written informed consent was provided by all patients before surgery. All patients underwent reconstructive lower lid surgery for dermatological neoplastic lesions using autologous chondral-perichondral graft tissue from the recipient auricular chonca. All patients underwent radical resection of the neoplastic lesion; intraoperative histological examinations of several sections were performed to ensure that lateral and medial resection margins were negative. The extent of tissue surgical removal ranged from half to four fifths of the eyelid length, which were always full-thickness excision.

Patients were followed by both surgical and ophthalmic specialists weekly for the first month, then monthly, to assess neoplasm reformation, surgical outcomes and possible complications related to the eyelid reconstruction.

### 2.2. Surgical Technique

**Figure 1:**
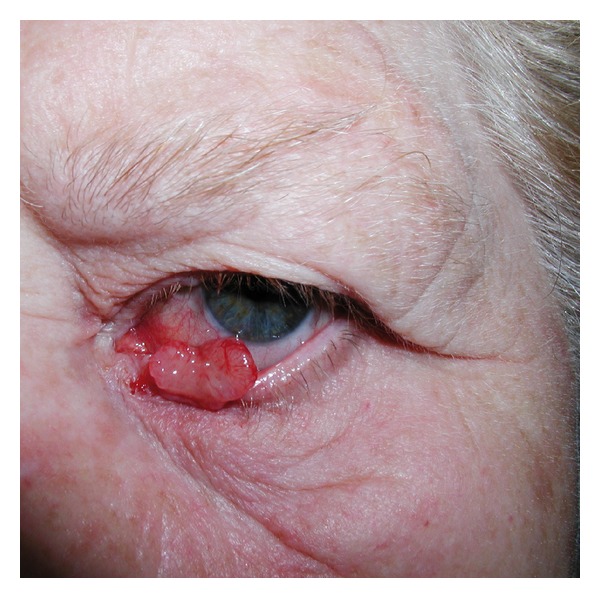
Patient with skin cancer on the left inferior lid before tumor resection.

The surgical procedure used in our department is based on the lower eyelid reconstruction technique with conchal cartilage graft described by Matsuo et al. [2, 3], however, with several modifications. In brief, the surgical removal of the lesion includes prepping the patient for general or local anesthesia with sedation; covering the bulb with a protective contact lens; excising the lower eyelid lesion to ensure macroscopically clear oncological margins (Figure 1); intraoperative histological assessment and further excision if needed. 

**Figure 2:**
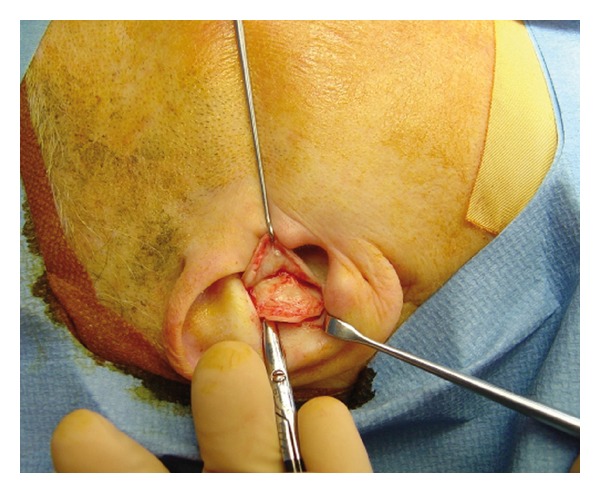
Intraoperative image shows the procedure to harvest the conchal cartilage.

The lid reconstruction is then commenced. The extent of lid tissue loss is assessed to determine the size of the cartilage grafted required. The inserted chondral graft should be slightly smaller than the defect to limit postoperative ectropion. With regards to the relative palpebral continence, the reduction should be approximately 3 mm but needs to be greater if the patient has major tarsal laxity or clinically manifest ectropion. The snap-back test is a simple and effective way to assess eyelid laxity. The graft tissue is usually taken from the auricle concha via an anterior access, by performing a curved 1.5–2.5 cm incision in the area found between the concha and the antihelix (Figure 2). The layer over the perichondrium is detached and a suitably sized portion of chondroperichondral concha is removed. It is imperative at this stage to keep the perichondral layer intact. After an accurately hemostasis, the auricular incision is sutured with Ethilon 5/0 or 6/0.

**Figure 3:**
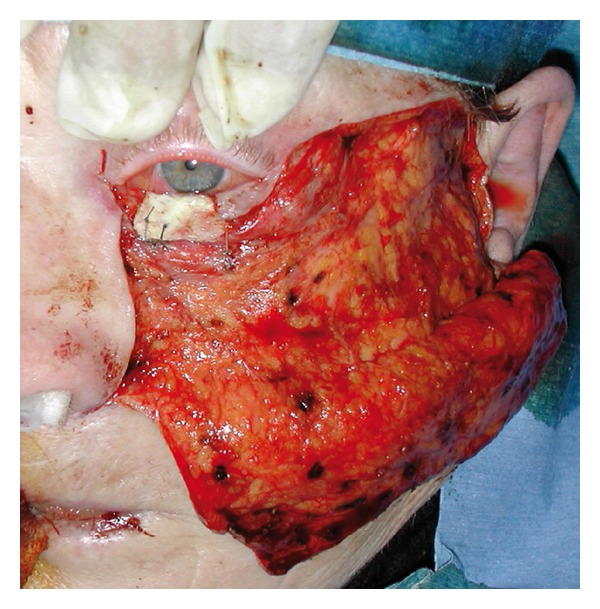
The graft is positioned with the perichondral side facing internal, so that it is in direct contact with the conjunctiva. The graft is then sutured to the tarsal stumps and residual eyelid conjunctiva with absorbable (Vicryl Rapid 7/0) stitches; the flap (Mustardé's flap) is harvested and ready to cover the graft and the soft tissue defect.

The cartilage graft is then carefully prepared for proper fitting. The graft thickness is decreased to adequate size and a series of surface incisions are made (in a careful manner to avoid damaging the perichondrium in accordance with Gibson's principle [4] in order to adapt the graft curvature to the convex surface of the eye). The perichondrium should extend at least 1-2 mm beyond the cartilage on all sides, in order to facilitate suturing it to the conjunctival plane. The graft is positioned with the perichondral side facing internally, so that it is in direct contact with the conjunctiva. The graft is then sutured to the tarsal stumps and residual eyelid conjunctiva with absorbable (Vicryl Rapid 7/0) stitches (Figure 3).

The lower eyelid is then reconstructed with either myocutaneous (mono- or bipedunculated Tripier) flaps, or cutaneous (Mustardé, McGregor) flaps [5]. The skin suture is then completed and a front suspension is prepared, which is left for 48–72 hours (providing that it does not cause excessive discomfort). Then the patient is medicated daily for the first 3 days and then on alternate days thereafter. The stitches are removed on the 6-7th postoperative day.

### 2.3. Follow-Up

The follow-up period for our cohort ranges from 6 to 36 months. All patients underwent a complete pre- and postoperative ophthalmologic examination, which included best-corrected visual acuity, slit-lamp examination, Goldmann applanation tonometry, and fundus biomicroscopy. Ophthalmological visits also provided a detailed and thorough assessment of the anterior surface, including pre- and postoperative lid margin and eyelash symmetry, clinical aspect of the cornea and conjunctiva, Schirmer test, and anterior segment color photos. The assessment of postoperative reconstruction quality was based on the following parameters: position of the eyelid, the closing capacity of lid rim, clinical manifestations of lagophthalmos or ectropion, surgeon's evaluation of esthetic surgical outcome, eyelid symmetry and morbidity of the graft donor area.

**Figure 4:**
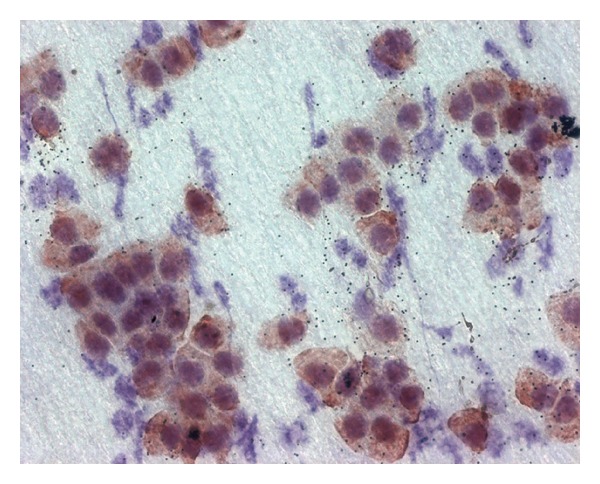
The reddish-brown cells show the presence of CK19 antigen on specimen cells, demonstrating and confirming newly formed conjunctiva overlying the graft.

Biopsies were taken after approximately a year and assessed with histopathology and cytology analysis. The analysis included impression cytology technique using antibodies against cytokeratin 19 (CK19) to confirm newly formed conjunctiva overlying the graft. Immunocytochemistry is a routine method commonly used in keratoplasty patients to assess ocular surface corneal and conjunctiva integrity. This technique was used in our cohort to confirm the presence of functional epithelium conjunctiva overlying the grafted perichondrium tissue. To obtain specimens, the ocular surface was anesthetized with Novesin 0.4% drops. After a few minutes, a 4 mm disc of filter membrane was placed on the internal side of the grafted tissue lid conjunctiva and a slight uniform pressure was applied. The specimen was peeled gently from the conjunctival surface and prepared for laboratory analyses. The procedure was then repeated to acquire a second specimen in the same eye. Immunocytochemistry staining was used to assess the presence of CK19 antigen on specimen cells, which appeared reddish-brown in color (Figure 4). Hepatic tissue was used as a normal control for CK19 immunostaining; biliary ducts and hepatocytes were used as positive and negative controls, respectively.

## 3. Results

**Figure 5:**
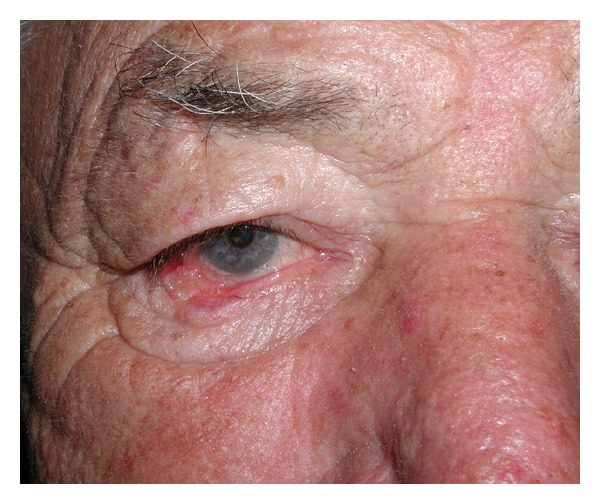
Preoperative views of skin cancer resection at right lower eyelid; reconstruction with conchal cartilage and Tripier flap.

**Figure 6:**
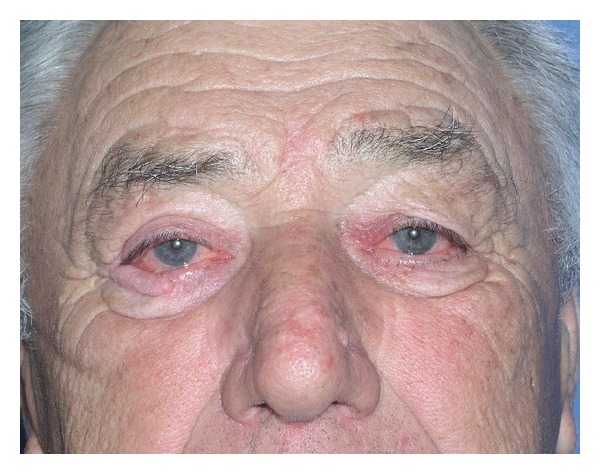
Postoperative views after 3 months in a patient with skin cancer resection at right lower eyelid; reconstruction with choncal cartilage and Tripier flap.

All the grafts adhered completely. None of the patients developed postoperative complications related to the inserted graft. There were only minor complications observed after surgery. There were no signs of ischemic on the overlying flap in all patients. All 20 patients showed no graft rejection on all follow-up controls, and surgical outcomes were generally good with minimal ocular complications. Sixteen of 20 patients had excellent results (mean follow-up 3 years), with good lid symmetry and esthetics, minimal auricular discomfort, patient satisfaction, and proper lid function. Figures 5 and 6 show a patient before and after surgery.

Surgical outcomes were highly dependent on proper postoperative conjunctiva formation. All patient specimens were CK19 antigen positive with impression cytology, thus indicating proper conjunctiva tissue formation. All patients showed a good to optimal capacity to close their eyelids completely, with no clinical signs of ectropion or lagophthalmos.

The symmetry between the two eyelids in all patients was optimal. The morbidity in the donor area was negligible, considering that the resulting cutaneous scar was perfectly hidden by the raised ear rim. All patients were generally happy with the functional and esthetic surgical outcomes, with patient satisfaction ranging from satisfactory to very pleased. 

Only one patient showed a short-term postoperative complication the day after surgery, in which a subpalpebral hematoma developed after the preparation of the monopedicled Tripier flap; this was surgically drained and later provided long-term functional and esthetic results. 

**Figure 7:**
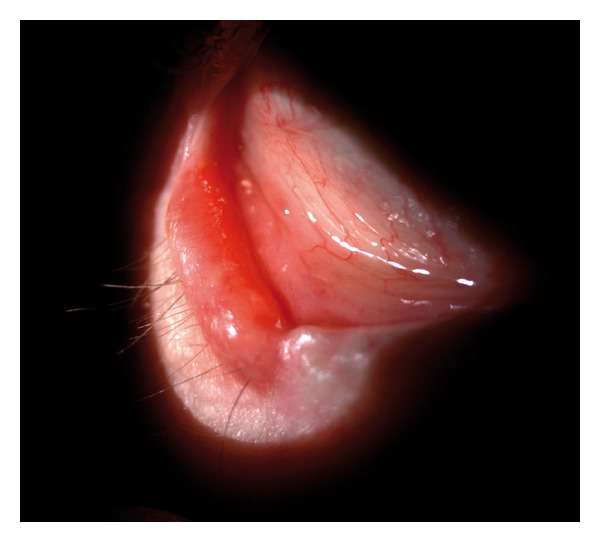
Granulation-like tissue within the conjunctival tissue underlying the graft perichondrium. The tissue was biopsied under topical anesthesia, and histological assessment of the specimen resulted as reactive granulation cells.

The long-term complications included cartilage protrusion from the rim of the lower eyelid in two patients, which caused no conjunctiva or cornea irritation and was later surgically corrected under local anesthesia. In addition, one patient developed excessive amounts of granulation-like tissue within the conjunctival tissue underlying the graft perichondrium (Figure 7). The tissue was biopsied under topical anesthesia, and histological assessment of the specimen resulted as reactive granulation cells. This minor complication was treated with local cortisone eye drops twice a day for 2 weeks, resulting in complete recovery.

## 4. Discussion

There have always been problems related to reconstruction eyelid surgery preceding full-thickness loss of substance, especially concerning compatible replacement tissue that can function as well as the missing tissue. The goal of this type of reconstructive surgery is to provide the lower eyelid with a support that is stable yet capable of adapting to the convexity of the eyeball. The reconstructed eyelid soft tissues (flaps or grafts) must adhere to the host tissue without giving rise to subsequent palpebral deformity (i.e., shrinkage due to scarring). The anatomical lower eyelid support is provided by the tarsus, and thus success in lower eyelid reconstruction is based on restoring tarsal continuity with suitable tissue.

Numerous tissues have been proposed, including grafts of fascia lata, cartilage from the nasal septum, chondromucosal flaps [6], grafts from the superior lateral nasal cartilage, tarsalconjunctival flaps from the upper eyelid [7], grafts of fascia temporalis and palate mucosa [8], and chondroperichondral grafts obtained from the cartilage of the concha of auricle [2, 3]. The standardized methods that have been used for lower eyelid reconstruction can be divided into three groups:

1. tarsal-conjunctival flaps of upper eyelid with dermal-epidermal grafts (Mustardé, Kollner, Llandolt-Hughes, and Hubner) [5, 9];
2. chondromucosal grafts or flaps coated with local skin flaps (Scuderi et al. [6] and Millard);
3. chondroperichondral grafts coated with local skin flaps [2, 3, 10, 11].

With regards to the first group, it is obvious that the ideal reconstruction involves replacing the missing tissue with another that is virtually identical. The morbidity to the upper eyelid becomes unacceptably severe, however, for surgery that involves replacing loss of substance exceeding a quarter to a third of the total length of the eyelid [5].

As for the second group, chondromucosal grafts and flaps are advantageous in that the mucosal tissue is positioned in direct contact with the conjunctival side of the eyelid and that the graft provides good stability to the eyelid's load-bearing structure. The disadvantages of these methods include the risk of failing to obtain suitable tissue from the nasal septum, iatrogenic septal perforation, technical difficulties in preparing the pedunculated flap from the superior lateral cartilage, and the presence of mucosal as opposed to conjunctival tissue on the posterior lamella of the eyelid.

The third group of reconstruction methods that utilizes chondro-perichondral grafts harvested from the concha of auricle offer the following advantages: low morbidity exists at the donor site; grafts are easy to obtain from the conchal tissue; the reconstruction does not involve complicated surgical techniques. Our previous study confirmed these advantages with this reconstruction technique, which reported good surgical functional and esthetic outcomes without major complication [12]. In our current study, there appeared to be a complete “restitutio ad integrum” of the conjunctiva after a short postoperative period in all our patients, which was shown by the cytology analysis. Our study adds to the literature in this field by providing an update and confirmation of our previous results by assessing surgical outcomes in a different cohort of patients, in addition to including data regarding several other ocular surface parameters and CK19 immunohistochemistry to confirm newly formed conjunctiva overlying the graft.

The conchal cartilage graft is easy to prepare so that it is adaptable to the convex surface of the eyeball and has shown to be an excellent tissue for supporting the lower eyelid, with limited palpebral rim deformity due to retraction induced by scarring of the locoregional skin flaps. Moreover, the perichondrium tissue aids in the proper progressive formation of conjunctiva overlying the inner surface of the graft that becomes evident within a few weeks, which has been reported in previous studies [13] and also been seen in all our patient biopsies. The normal expression of cytokeratin CK19 in the eyelid supports the use of the CK19 immunohistochemistry technique to prove the formation of new conjunctiva [14]. 

It is important to note (especially in elderly patients that often have transversal eyelid laxity) that the width of the graft needs to be accurately measured and should be at least 3 mm smaller than the dimensions of the eyelid defect in order to limit postoperative ectropion. Once the graft tissue has been obtained, it is extremely important to prepare and shape it correctly. The cartilage conchal should be coated on the inside with an intact perichondrium, which should extend at least 1-2 mm beyond the cartilage on all sides in order to facilitate adhesion to the residual conjunctiva and the skin of the transposed flap. This technical detail ensures proper bulbar conjunctiva and cornea protection and enables a more rapid migration of the adjacent conjunctiva under the regenerative guidance of the perichondrium. On the contrary, areas of conchal cartilage that are not coated with perichondrium or perichondrium that does not properly adhere to residual cartilage show early signs of reactive granulation tissue developing on the internal lining. The conchal cartilage is considerably thicker than that of the tarsal lamina, thus providing adequate stiffness and support to the reconstructed eyelid. This feature was seen in patients first undergoing this procedure, which showed signs of good lower eyelid fixity, however, with slight difficulty in gaze fixation downwards (albeit well tolerated in our patients). The conchal cartilage should thus be thinned out to the required thickness by tangential sectioning of the graft using a no. 11 scalpel. The graft can easily be prepared in this manner under a magnifying lens, which should be shaped to ensure good foldability and adaptation to the eyeball curvature. Foldability can also be improved by vertically scoring the cartilaginous side of the graft, based on Gibson's cartilage modeling principles.

The main drawback of this technique is due to the initial irritation of the bulbar conjunctiva that is in contact with the perichondral graft tissue, which may cause some discomfort during the first few days after surgery. This problem spontaneously resolves with time in a matter of a few weeks, thanks to the proliferation of the conjunctiva that is properly oriented by the perichondrium. The surgeon needs to work in collaboration with the ophthalmologist during the pre- and postoperative follow-ups to ensure long lasting successful outcomes.

In closing, our experience with the reconstructive technique involving chondro-perichondral graft harvested from the auricle concha shows that it provides successful postoperative outcomes, with functional and esthetically pleasing reconstructed lower eyelid. Postoperative complications and long-term side effects tend to be minimal. Autologous chondral-perichondral tissue provides good functional and mechanical support in the reconstructed lid, thus reducing the risks of ectropion and corneal exposure and ensuring a protected ocular surface. Moreover, the direct contact of perichondral tissue with the bulbar conjunctiva aids in new conjunctival formation of the reconstructed portion of the lid, thus minimizing ocular complications.
